# Galectin-3, metalloproteinase-2 and cardiovascular disease were independently associated with metalloproteinase-14 in patients with type 1 diabetes: a cross sectional study

**DOI:** 10.1186/s13098-021-00727-3

**Published:** 2021-10-26

**Authors:** Eva Olga Melin, Jonatan Dereke, Magnus Hillman

**Affiliations:** 1 Department of Clinical Sciences, Diabetes Research Laboratory, Faculty of Medicine, Lund University, Lund, Sweden; 2 Department of Research and Development, Region Kronoberg, Växjö, Sweden

**Keywords:** Antidepressants, Biomarkers, Cardiovascular disease, Depression, Metalloproteinases, Tissue inhibitors of metalloproteinases, Type 1 diabetes

## Abstract

**Background:**

Type 1 diabetes (T1D) is a major risk factor for cardiovascular disease (CVD). Matrix metalloproteinase-14 (MMP-14) is involved in the development of atherosclerosis and CVD. The main aim was to explore the associations between MMP-14 and selected inflammatory and metabolic variables, CVD, depression, physical activity, smoking and medication in patients with T1D. The secondary aim was to explore associations with CVD.

**Methods:**

Cross-sectional design. The participants were consecutively recruited from one specialist diabetes out-patient clinic. Depression was assessed by a self-report instrument. Blood samples, anthropometrics and blood pressure were collected, supplemented with data from electronic health records. High MMP-14 was defined as  ≥  5.81 ng/mL. Non-parametric tests, Chi^2^ tests and multiple logistic regression analyses were performed.

**Results:**

Two hundred and sixty-eighth T1D patients aged 18–59 years participated (men 58%, high MMP-14 25%, CVD 3%). Sixty-seven patients with high MMP-14, compared to 201 patients with lower MMP-14, had higher prevalence of CVD (8% versus 1%, *p*  =  0.012), and had higher levels of galectin-3 (*p * <  0.001) and MMP-2 (*p * =  0.018). Seven patients with CVD, compared to 261 without, were older (*p*  =  0.003), had longer diabetes duration (*p*  =  0.027), and had higher prevalence of high MMP-14 (71% versus 24%, *p*  =  0.012), abdominal obesity (*p*  =  0.014), depression (*p * =  0.022), usage of antidepressants (*p*  =  0.008), antihypertensive drugs (*p*  =  0.037) and statins (*p*  =  0.049).

Galectin-3 (per ng/mL) [adjusted odds ratio (AOR) 2.19, *p*  <  0.001], CVD (AOR 8.1, *p*  =  0.027), and MMP-2 (per ng/mL) (AOR 1.01, *p * =  0.044) were associated with high MMP-14. Depression (AOR 17.4, *p * =  0.006), abdominal obesity (15.8, *p*  =  0.006), high MMP-14 (AOR 14.2, *p*  =  0.008), and diabetes duration (AOR 1.10, *p*  =  0.012) were associated with CVD.

**Conclusions:**

The main findings of this study were that galecin-3, MMP-2, and CVD were independently associated with high levels of MMP-14 in T1D patients. The association between MMP-14 and galectin-3 is a new finding. No traditional risk factors for CVD were associated with MMP-14. Depression, abdominal obesity and MMP-14 were independently associated with CVD.

## Background

Type 1 diabetes (T1D) is a major risk factor for cardiovascular disease (CVD) [1, 2]. The risk for developing CVD in people with T1D is increased by early onset, female sex, metabolic and inflammatory disturbances, unhealthy behaviour, depression, and chronic kidney disease [1–6].

Metalloproteinases (MPs) are vital for extracellular matrix (ECM) remodelling and degradation activities in multiple organ systems such as the cardiovascular (CV) and central nervous systems, as well as the kidneys [7–12]. MPs target a wide range of substrates, are involved in inflammatory and immune responses, and are regulated by their endogenous inhibitors—the tissue inhibitors of metalloproteinases (TIMPs) [7–12].

Several MPs are involved in the development of atherosclerosis which may lead to severe CVD such as ischemic heart disease, heart failure, stroke, and death [7–9, 11, 12]. Matrix metalloproteinases (MMPs) are expressed in atherosclerotic plaques, promoting vascular remodelling, contributing to atherothrombosis, and plaque disruption [9, 12–14]. They are also involved in cardiac remodelling after myocardial infarction, leading to dilated cardiomyopathy [9]. MMP-14, is a type 1 transmembrane proteinase (MT1-MMP) which modifies both the pericellular microenvironment in the ECM and the cell function, and is inhibited by TIMP-2 and TIMP-3 [15]. MMP-14 and TIMP-2 are both necessary for the activation of pro-MMP-2 [15]. MMP-14 has been confirmed in human atherosclerotic plaques and in cardiomyocytes [7, 13, 14]. It has been demonstrated that MMP-14 is upregulated in a particularly harmful macrophage phenotype (MMP14^+^TIMP3^−^), which contributes to increased risk for plaque rupture and myocardial infarction [14]. MMP-14 is also upregulated post-infarction contributing to cardiac remodelling [16]. Decreased levels of TIMP-3 have been linked to ventricular remodelling after myocardial infarction [17].

Increased plasma levels of MMP-2 have been demonstrated in people with T1D compared to non-diabetic controls [18]. MMP-2 has been linked to CVD and all-cause mortality [12, 19]. MMP-2 was in a previous study of heart failure correlated with Galectin-3 [20], which in turn previously was linked to atherosclerosis and CVD [21]. Increased MMP-9 levels have been linked to CVD [19, 22, 23]. Both increased levels of MMP-2 and MMP-9 stimulate the rupture of vulnerable arterial plaques [7]. Several cardiovascular risk factors, such as hyperglycemia, increased serum-lipids, and smoking, contribute to oxidative stress or the formation of advanced glycation end products, which in turn promote the activation of MMPs [9, 24].

We hypothesize that MMP-14 in a complex manner is involved in the development of CVD. The main aim was to explore theQuery associations between MMP-14 and selected inflammatory and metabolic variables, CVD, depression, physical activity, smoking, and medication, in patients with T1D. The secondary aim was to explore variables associated with CVD.

## Materials and methods

### Participants and study design

The study has a cross sectional design and included 268 (65%) patients out of 415 eligible patients with T1D. Inclusion criteria were T1D patients aged 18–59 years, diabetes duration  ≥  1-year, and performed measurements of MMP-14. Exclusion criteria were pregnancy, severe somatic and psychiatric disorders such as cancer, hepatic failure, end-stage renal disease, Cushing’s disease, severe autoimmune disorders, psychotic, bipolar or severe personality disorders, severe substance abuse, cognitive deficiency (due to stroke, dementia or intellectual disability), use of systemic corticosteroids, and inadequate knowledge of the Swedish language. The patients were consecutively recruited during a 9-month period, from 25 March to 28 December 2009, from the largest out of two hospital diabetes outpatient clinics in Region Kronoberg, Sweden. The catchment population was 1,25,000. A self-report questionnaire was used to assess depression. Blood samples, anthropometrics and blood pressure were collected, supplemented with data from electronic health records.

### Cardiovascular disorder

CVD was defined as ischemic heart disease [angina pectoris, previous myocardial infarction, performed percutaneous transluminal coronary angioplasty (PTCA), and/or coronary artery bypass graft (CABP) surgery], heart failure, stroke, and/or transient ischemic attack (TIA).

### Depression

Self-reported depression was assessed by Hospital Anxiety and Depression Scale- the depression subscale (HADS-D), and was defined as HADS-D  ≥  8 points [25, 26].

### Thyroid disease

Patients treated for hypothyroidism or hyperthyroidism were identified, but not excluded.

### Biochemical analyses

Plasma levels of MMP-2, MMP-14, TIMP-2, and TIMP-3, were analysed by using commercial human DuoSet enzyme linked immunosorbent assays (ELISAs) and supplementary ancillary kit (R&D Systems, Minneapolis, MN, USA). Before the analyses, the plasma samples were diluted in phosphate-buffered saline (PBS) supplemented with 1% bovine serum albumin (BSA), and were run in duplicates. The dilution factors were for MMP-2: 1:30; MMP-9: 1:400; MMP-14: 1:3 (1:25/1:50); TIMP-2: 1:400; TIMP-3: 1:16; and Galectin-3: 1:2. The ELISA analyses were performed according to the manufacturer’s instructions. Absorbance was measured at 450–580 nm in a FLOUstar optima plate reader (BMG Labtech Gmbh, Ortenberg, Germany). Concentrations of unknown samples were calculated using a 4-parameter logistic regression curve. The intra-assay coefficients of variation were for MMP-2: 3.7%; MMP-9: 2.2%; MMP-14: 2.8%; TIMP-2: 2.0%; TIMP-3: 1.6%; and galectin-3: 4.3%. The analyses were performed at the Diabetes Laboratory, BMC, Lund University, Lund.

For MMP-14 there were exact values for 173 patients, but for 95 patients the MMP-14 values were under the detection limit of 0.154 ng/mL. The undetected values were approximated by “zero”.

Hemoglobin A1c (HbA1c) and serum (s)-lipids were collected after overnight fasting and analysed with an Olympus automated clinical chemistry analyser with high specificity (Olympus AU^®^, Tokyo, Japan). The intra-coefficients of variation were for HbA1c  <  1.2%; total cholesterol  <  2.1%; High-density lipoprotein (HDL)-cholesterol  <  3.0%; low-density lipoprotein (LDL)-cholesterol  <  2.6%; and for triglycerides  <  2.2%.

S-creatinine was assayed by an AU2700^®^ instrument (Beckman Coulter, Brea, CA, USA). The intra-coefficient of variation was  <  3%. HbA1c, s-lipids and s-creatinine were analysed at the department of Clinical Chemistry, Växjö Central Hospital.

CRP was assayed by spectrophotometry on a Roche Cobas C501^®^ at the diabetes laboratory, Lund University Hospital, Lund.

### Anthropometrics and blood pressure

Waist circumference, weight and length were measured by a nurse. Body mass index (BMI) (kg/m^2^) was calculated. Abdominal obesity was defined as WC  ≥  1.02 m for men and as WC  ≥  0.88 m for women [27]. Blood pressure was measured according to standard procedures in the sitting position by a nurse.

### Smoking and physical activity

Smokers were defined as having smoked any amount of tobacco during the last year [27]. Information regarding levels of physical activity was collected by interviews performed by skilled nurses or physicians. Five levels were initially registered as in the Swedish National Diabetes Register (S-NDR) [28]:  ≥  30 min of moderate activities were performed either (1) never, (2) less than once a week, (3) 1–2 times a week, (4) 3–5 times a week, or (5) daily. In this study, level 1 and 2 were merged into one level: less than once a week.

### Treatment for T1D

The patients used either multiple daily insulin injections (MDII) or continuous subcutaneous insulin infusion (CSII) administered by a pump.

### Antihypertensive drugs and indications for treatment of hypertension

Antihypertensive drugs included ACE inhibitors (ATC codes C09AA-BA); (ARB) (ATC codes C09CA-DA); calcium antagonists (ATC codes C08CA01-02); diuretics (ATC codes C03AA03 or C03CA01); and/or selective beta-adrenoreceptor antagonists (ATC code C07AB). Indications for antihypertensive drugs were systolic BP  >  130 mm Hg and/or BP  >  80 mm Hg and/or CVD according to the Swedish national guidelines in 2009 [29]. The use of antihypertensive drugs was dichotomized into users and non-users.

### Lipid-lowering drugs and indications for treatment of hyperlipidemia

Lipid-lowering drugs were hydroxy-methylglutaryl coenzyme A (HMG-CoA) reductase inhibitors (statins) (ATC-code C10AA). Indications for lipid-lowering drugs were total cholesterol  >  4.5 mmol/L (>  1.74 mg/dL) and/or LDL-cholesterol  >  2.5 mmol/L (>  97 mg/dL) or present CVD according to the Swedish national guidelines in 2009 [29]. The use of lipid-lowering drugs was dichotomized into users and non-users.

### Antidepressants

Antidepressants were SSRIs, SNRIs and/or specific serotonergic antidepressants (N06AB, N06AX16, or N06AX11). The use of antidepressants was dichotomized into users and non-users.

### Statistical analysis

Analysis of data distribution using histograms revealed that age, diabetes duration, and the inflammatory plasma biomarkers were not normally distributed. Data were presented as median [quartile (q)_1_, q_3_; min–max], and analyses were performed with Mann–Whitney *U* test. Fisher’s Exact Test (two-tailed) and Linear-by-linear association were used to analyse categorical data, and data were presented as N (%). Medians and prevalence rates for the included variables were compared between patients with high MMP-14 (≥  5.81 ng/mL) and low MMP-14 (<  5.81 ng/mL), and between patients with and without CVD.

Variables with *P *values  <  0.10 were included in the further analyses and crude odds ratios (CORs) were calculated. Variables with *P *values  <  0.10 for the CORs were included in multiple logistic regression analyses (Backward: Wald) with high MMP-14 (one model) and CVD (three models) as dependent variables. In the first model, only baseline variables were included. In the second model, variables with P values  <  0.10 in model 1 and MMP-14 used as a continuous variable were included. In the third model, continuous MMP-14 was exchanged by high MMP-14, otherwise the variables were the same as in model 2. The Hosmer and Lemeshow test for goodness-of-fit and Nagelkerke R2 were used to evaluate each multiple logistic regression analysis model. The area under the receiver operating characteristics curve (AUC of ROC) was performed for MMP-14 tried against CVD, in order to establish a cut-off value for MMP-14, based on the combined optimal sensitivity and specificity. To evaluate the statistical power, a post-hoc calculation was performed for high MMP-14 and CVD and showed a power of 79.7%: the result was calculated using the following data: group 1, 7 patients had CVD and the prevalence of high MMP-14 was 71%; group 2, 261 patients did not have CVD and the prevalence of high MMP-14 was 24% [30]. *P* values  <  0.05 were considered statistically significant. SPSS^®^ version 25 (IBM, Chicago, Il, USA) was used.

## Results

In the study 268 patients with T1D (aged 18–59 years, 58% men) participated. Nine percent used CSII and 91% used MDII.

The AUC (CI 95%) for MMP-14 tried against CVD was 0.75 (0.55–0.95), *p*  =  0.023, and the cut-off value MMP-14  ≥  5.81 ng/mL corresponded to a sensitivity of 0.71 and specificity of 0.70. MMP-14  ≥  5.81 ng/mL corresponded to the 75th percentile.

In Table 1, baseline characteristics are compared between patients with and without high levels of MMP-14 (≥  5.81 ng/mL), and between patients with and without CVD. The 67 patients with high MMP-14, compared to the 201 patients with low MMP-14, had a higher prevalence of a CVD (*p*  =  0.012). The 7 T1D patients with CVD, compared to the 261 patients without CVD, were older (*p*  =  0.001), had longer diabetes duration (*p*  =  0.027), and had higher prevalence of abdominal obesity (*p * =  0.014), depression (*p*  =  0.022), and use of antihypertensive drugs (*p*  =  0.037), statins (*p * =  0.049), and antidepressants (*p*  =  0.008).

**Table 1:** Baseline variables presented for patients with and without cardiovascular disease

	All patients	High MMP-14 (≥ 5.81 ng/mL)			Cardiovascular disease		
		Yes	No	*P*^a^	Yes	No	*P*^a^
*N*	268	67	201		7	261	
Age (years)	(18–59)	42 (32, 48)	42 (31, 52)	0.60	55 (46, 59)	42 (31, 50)	0.003
Diabetes duration (years)	(1–55)	21 (9, 31)	20 (11, 30)	0.97	35 (19, 44)	20 (10, 30)	0.027
Sex							
Men	154 (58)	42 (63)	112 (56)	0.39^b^	4 (57)	150 (58)	1.00^b^
Women	114 (42)	25 (37)	89 (44)		3 (43)	111 (42)	
Abdominal obesity^c^	46 (16)	10 (15)	32 (16)	1.00^b^	4 (57)	38 (15)	0.014^b^
BMI (kg/m^2^)^d^	(18–45)	25 (23, 27)	25 (23, 28)	0.72	23 (22, 33)	25 (23, 27)	0.63
Systolic blood pressure (mm Hg)	(90–160)	125 (115, 130)	110 (120, 130)	0.21	120 (120, 135)	120 (110, 130)	0.38
Diastolic blood pressure (mm Hg)	(55–100)	70 (70, 78)	70 (68, 75)	0.40	75 (70, 75)	70 (70, 75)	0.64
Physical activity^e^							
Daily	93 (37)	27 (42)	66 (35)	0.99^f^	1 (14)	92 (37)	0.82^f^
3–5/week	80 (31)	19 (29)	61 (32)		4 (57)	76 (31)	
1–2/week	54 (21)	13 (20)	41 (22)		2 (29)	52 (21)	
< 1/week	27 (11)	6 (9)	21 (11)		0	27 (11)	
Smoking^g^	28 (11)	7 (11)	21 (11)	1.00	1 (14)	27 (11)	> 0.56^b^
CVD	7 (3)	5 (8)	2 (1)	0.012^b^	–	–	–
Depression	26 (10)	8 (12)	18 (9)	0.48^b^	3 (43)	23 (9)	0.022^b^
Thyroid disease^h^	28 (11)	4 (6)	24 (12)	0.25^b^	0	28 (11)	1.00^b^
CSII^i^ (users)	23 (9)	5 (8)	18 (9)	0.81^b^	0	23 (9)	1.00^b^
Antihypertensive drugs (users)	86 (32)	18 (27)	68 (34)	0.36^b^	5 (71)	81 (31)	0.037^b^
Statins (users)	122 (46)	30 (45)	92 (46)	1.00^b^	6 (86)	116 (44)	0.049^b^
Antidepressants (users)	18 (7)	6 (9)	12 (6)	0.37^b^	3 (43)	15 (6)	0.008^b^

Data are presented as N (%), (min–max) or median (q_1_, q_3_)

^a^Mann–Whitney *U* test unless indicated

^b^Fisher’s Exact Test

^c^5; ^d^2; ^e^14: missing values (N)

^f^Linear-by-linear association

^g^15; ^h^9: missing values (N)

^i^Patients not using CSII are using MDII

In Table 2, the results of all biochemical analyses are compared between patients with and without high levels of MMP-14, and between patients with and without CVD. The patients with high MMP-14 levels had higher levels of MMP-2 (*p*  =  0.018) and galectin-3 (*p * <  0.001). The patients with CVD had higher median levels of MMP-14 (*p*  =  0.020) and TIMP-2 (*p*  =  0.028). The prevalence of high MMP-14 was three times higher in patients with CVD compared to patients without CVD (71% versus 24%, *p*  =  0.012).

**Table 2:** Biochemical analyses compared between people with high and low MMP-14 and between people with and without CVD

	All patients	High MMP-14 (≥ 5.81 ng/mL)			Cardiovascular disease		
	268	Yes	No	*P*^a^	Yes	No	*P*^a^
		67	201		7	261	
MMP-14 (ng/mL)	(0.00–447.0)	18.4 (10.2, 42.1)	0.65 (0.0, 1.92)	< 0.001	12.2 (2.1, 95.3)	1.5 (0.0, 5.4)	0.020
MMP-14 ≥ 5.81 (ng/mL)	67 (25)	–	–	–	5 (71)	62 (24)	0.012^b^
MMP-2 (ng/mL)	(65.7–434.0)	164.0 (135.0, 197)	150 (128.5, 175.5)	0.018	176.0 (162.8, 217.5)	154.0 (129.8, 177.2)	0.16
MMP-9 (ng/mL)	(222–1936)	515 (400, 637)	518 (408–720)	0.58	518 (447–835)	515 (405–702)	0.55
TIMP-2 (ng/mL)^c^	(0.00–468.0)	187.0 (161.0, 213.0)	174.5 (151.0, 202.8)	0.060	205.0 (195.0, 263.0)	178.0 (152.5, 205.0)	0.028
TIMP-3 (ng/mL)^d^	(1.2–692.3)	8.2 (5.9, 11.0)	8.4 (5.8, 12.0)	0.71	9.2 (6.3, 13.1)	8.2 (5.8, 11.4)	0.40
Galectin-3 (ng/mL)^e^	(0.001–36.4)	1.9 (1.0, 3.5)	0.8 (0.5, 1.4)	< 0.001	1.7 (0.6–3.2)	0.9 (0.6–1.7)	0.44
CRP (mg/L)^f^	(0.03–8.4)	0.6 (0.2, 1.9)	0.7 (0.3, 1.7)	0.72	0.6 (0.4, 5.0)	0.6 (0.2–1.6)	0.40
HbA1c							
(mmol/mol)	(25–110)	58 (53, 67)	63 (54, 71)	0.054	65 (51, 74)	63 (54, 71)	0.94
(%)	(4.4–12.2)	7.5 (7.0, 8.3)	8.0 (7.1, 8.7)		8.1 (6.8, 8.9)	7.9 (7.1, 8.6)	
Total cholesterol (mmol/L)	(2.1–10.9)	4.7 (4.1, 5.2)	4.5 (4.1, 5.1)	0.53	4.0 (3.6, 5.0)	4.6 (4.1, 5.1)	0.12
LDL-cholesterol (mmol/L)	(0.6–8.3)	3.0 (2.5, 3.4)	2.7 (2.4, 3.3)	0.16	2.1 (1.9, 3.2)	2.8 (2.4, 3.3)	0.063
HDL-cholesterol (mmol/L)	(0.3–2.7)	1.4 (1.2, 1.8)	1.3 (1.5, 1.8)	0.44	1.7 (1.3, 2.0)	1.5 (1.3, 1.8)	0.44
Triglycerides (mmol/L)	(0.06–5.9)	1.0 (0.7, 1.3)	0.8 (0.7, 1.2)	0.102	1.0 (0.6, 1.5)	0.9 (0.7, 1.2)	0.88
S-creatinine (mmol/L)^g^	(28–182)	70 (64, 79)	70 (62, 78)	0.62	70 (64, 80)	70 (62, 78)	0.95

Data are presented as N (%), (min–max) or median (q_1_, q_3_)

^a^Mann–Whitney *U* test unless indicated

^b^Fisher’s Exact Test

^c^1; ^d^7; ^e^4; ^f^78; ^g^11: missing variables

In Table 3, associations with high MMP-14 are presented. CVD (adjusted odds ratio (AOR) 8.1, *p*  =  0.027), MMP-2 (per ng/mL) (AOR 1.01, *p*  =  0.044), and galectin-3 (per ng/mL) (AOR 2.19, *p*  <  0.001) were associated with high MMP-14 levels.

**Table 3:** Associations with high MMP-14

	High MMP-14 (≥ 5.81 ng/mL)			
	COR (CI 95%)	*P *value	AOR (CI 95%)	*P* value^a^
CVD	8.0 (1.5–42)	0.014	8.1 (1.3–52.5)	0.027
MMP-2 (per ng/mL)	1.01 (1.01–1.01)	0.016	1.01 (1.00–1.01)	0.044
TIMP-2 (per ng/mL)	1.00 (1.00–1.01)	0.13	1.00 (0.99–1.01)	0.92
Galectin-3 (per ng/mL)	2.15 (1.63–2.82)	< 0.001	2.19 (1.65–2.90)	< 0.001
HbA1c (mmol/mol)	0.98 (0.96–1.00)	0.054	0.98 (0.95–1.00)	0.10

^a^Multiple logistic regression analysis: backward (Wald): N  =  263; Nagelkerke R Square: 0.320; Hosmer and Lemeshow Test: 0.630

In Table 4, associations with CVD are presented for three models. In model 1, diabetes duration (AOR 1.10, *p*  =  0.016) and abdominal obesity (AOR 10.2, *p*  =  0.011) were associated with CVD. In model 2, diabetes duration (AOR 1.13, *p*  =  0.008), abdominal obesity (24.5, *p*  =  0.004), depression (AOR 22.1, *p*  =  0.006), and MMP-14 (per ng/mL) (AOR 1.01, *p * =  0.005), were associated with CVD. In model 3, diabetes duration (AOR 1.10, *p*  =  0.012), abdominal obesity (15.8, *p * =  0.006), depression (AOR 17.4, *p*  =  0.006), and high MMP-14 (AOR 14.2, *p*  =  0.008), were associated with CVD.

**Table 4:** Associations with CVD presented for three models

Cardiovascular disease								
	COR (CI 95%)	*P *value	Model 1^a^		Model 2^b^		Model 3^c^	
			AOR (CI 95%)	*P *value^a^	AOR (CI 95%)	*P *value^b^	AOR (CI 95%)	*P *value^c^
Age (per year)	1.16 (1.03–1.30)	0.016	1.10 (0.98–1.25)	0.12	–	–	–	–
Diabetes duration (per year)	1.10 (1.014–1.15)	0.016	1.10 (1.02–1.18)	0.016	1.13 (1.03–1.24)	0.008	1.10 (1.02–1.18)	0.012
Abdominal obesity	7.6 (1.6–35.5)	0.009	10.2 (1.7–62)	0.011	24.5 (2.8–217)	0.004	15.8 (2.2–113)	0.006
Depression	7.8 (1.6–36.8)	0.010	5.9 (0.9–40)	0.069	22.1 (2.4–201)	0.006	17.4 (2.2–135)	0.006
Antihypertensive drugs (users)	5.6 (1.1–29.2)	0.043	1.7 (0.2–13.6)	0.63	–	–	–	–
Statins (users)	7.5 (0.9–63.2)	0.06	3.0 (0.2–37)	0.40	–	–	–	–
Antidepressants (users)	12.3 (2.5–60.0)	0.002	5.0 (0.8–32)	0.090	4.8 (0.7–33)	0.12	4.6 (0.6–35)	0.13
MMP-14 (per ng/mL)	1.01 (1.00–1.01)	0.060	–	–	1.01 (1.00–1.02)	0.005	–	–
High MMP-14 (≥ 5.81 ng/mL)	8.0 (1.5–42.4)	0.014	–	–	–	–	14.2 (2.0–101)	0.008
TIMP-2 (per ng/mL)	1.01 (1.00–1.02)	0.102	–	–	–	–	–	–
LDL-cholesterol (per mmol/L)	0.3 (0.1–1.2)	0.085	0.5 (0.1–2.4)	0.38	–	–	–	–

Nagelkerke R Square: ^a^0.360/^b, c^0.452; Hosmer and Lemeshow Test: ^a^0.533/^b, c^0.956

^a–c^Multiple logistic regression analysis: backward (Wald): N  =  ^a, b, c^263

^a^No biochemical variables are included

^b^Variables with *P *values  <  0.10 in model 1 and MMP-14 used as a continuous variable were included

^c^Continuous MMP-14 was exchanged by high MMP-14, otherwise the variables were the same as in model 2

## Discussion

**Fig. 1:**
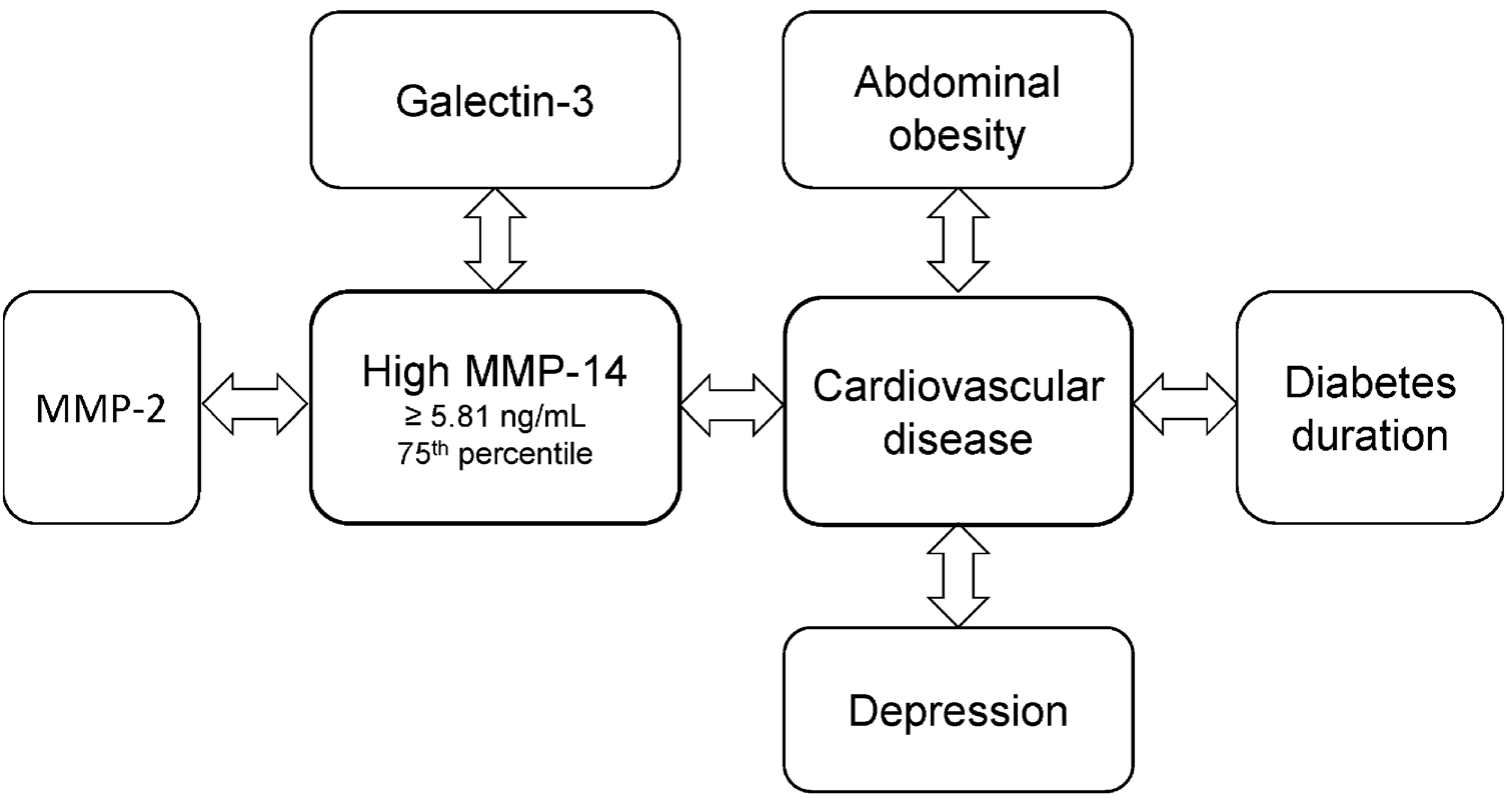
Illustration of the associations with high MMP-14 and CVD which were found in the study

The primary findings of this study of 268 adult patients with T1D were that galectin-3, CVD and MMP-2 were independently associated with high levels of MMP-14 (≥  5.81 ng/mL). Second, depression, abdominal obesity, high MMP-14, and diabetes duration, were independently associated with CVD (Fig. 1).

High levels of MMP-14 were associated with CVD, which is in line with previous research showing that MMP-14 is involved in the development of atherosclerosis with consequent cardiovascular disease [7, 13, 14, 16]. The demonstrated association between MMP-14 and MMP-2 is important as MMP-14 together with TIMP-2 enables the activation of pro-MMP-2 [15]. According to previous research, activated MMP-2 is another contributor to the development of CVD [12, 19], and increased MMP-2 levels have been demonstrated in patients with T1D [18]. Galectin-3, previously likewise linked to CVD [21], was associated with MMP-14, which to our knowledge is a new finding. Galectin-3 was previously linked to MMP-2 [20]. The strength of the association between high levels of MMP-14 and CVD was similar to the strengths of the associations between abdominal obesity, depression and CVD. Abdominal obesity is since long a well-established risk factor for atherosclerosis, coronary artery disease, stroke, and heart failure [6, 31]. The awareness of depression as a risk factor for CVD disease and mortality is lower, but clearly demonstrated in previous research [3].

To explore biomarkers is important for several reasons. First, identification of substances involved in the development of disease may lead to the development of new therapeutics [7]. In addition, biomarkers may be used for diagnosing CVD or evaluation of CVD risk [9, 32]. None of the traditional risk factors for CVD such as low grade inflammation (increased CRP), increased HbA1c, dyslipidaemia, increased blood pressure, physical inactivity, smoking, or the less established risk factor—depression, was associated with MMP-14 [1–3, 5]. The lack of associations between MMP-14 and the traditional risk factors makes MMP-14 a potentially interesting addition to traditional risk assessments.

Strengths of our study are that patients with severe comorbidities such as cancer, severe autoimmune disorders, hepatic failure, end-stage renal disease, psychotic and bipolar disorders were excluded as these disorders, or medication for these disorders, may have impact on the immune system including MMPs and galectin-3 [7, 33]. We explored and adjusted for relevant variables previously linked to CVD: MMP-2, MMP-9, MMP-14, TIMPS, galectin-3, CRP, metabolic variables, life style variables, depression, and thyroid disease [2, 3, 7–9, 11–14, 16, 17, 19, 21–23, 34–36]. The logistic regression models were elaborated for the associations, and calibrated and validated for goodness of fit with the data variables. By using a cut-off value for MMP-14, the problem with the very low and therefore unmeasurable MMP-14 levels was eliminated. Finally, precise ELISA techniques were used and the analyses showed low intra-assay coefficients of variation for all included plasma biomarkers.

Causality cannot be confirmed due to the cross-sectional design, which is a limitation. Another limitation was the restricted number of patients with CVD. The low prevalence of CVD was most likely due both to the exclusion of patients with end-stage renal disease [4], and the exclusion of patients with cognitive deficiencies induced by stroke or other causes of dementia. Despite the limited number of patients with CVD, the AUC was significant for MMP-14 tried against CVD. The post-hoc analyses of the power for the association between high MMP-14 and CVD was acceptable. However, the large CI intervals for high MMP-14 in the multiple logistic regression analyses adds uncertainty to the results, and shows that the results have to be tried and confirmed in a larger population of T1D patients with CVD. Due to the large CI intervals, continuous MMP-14 was tried against CVD which showed clear significance without broad CI intervals. A third limitation was that we did not identify patients suffering from peripheral artery disease. We cannot exclude that the levels of MMP-14 are influenced by the presence of a potential peripheral artery disease. A fourth limitation was that depression was only assessed by a self-report instrument, not by a structured interview. HADS has, however, shown high validity for assessing symptoms of depression both at an individual and a collective level in previous research [25]. Fifth, the levels of physical activity were not objectively measured, but data collected and categorized as in the S-NDR has shown strong associations between low physical activity and CVD [28]. Sixth, we have no information regarding dietary habits.

In future research we suggest evaluation of MMP-14 as a risk marker in larger and longitudinal studies in addition to conventional risk factors for CVD, and potentially in addition to other new biomarkers of CVD [32]. We also suggest further exploration of causes and mechanisms of the demonstrated association between MMP-14 and galectin-3.

## Conclusions

The main findings of this study of 268 patients with T1D were that galecin-3, MMP-2, and CVD were independently associated with high levels of MMP-14. The association between MMP-14 and galectin-3 is a new finding. No traditional risk factors for CVD in T1D patients were associated with MMP-14. Depression, abdominal obesity and high MMP-14 levels were independently and to an almost equal degree associated with CVD.

## Data Availability

The data set analyzed during the current study is not available publicly as individual privacy could be compromised, and we have no permission from the Regional Ethical Board to share the research data publicly. The data set is stored at the department for Research and Development, Region Kronoberg, Växjö, Sweden, and is available from the corresponding author on reasonable request.

## Abbreviations

AOR: Adjusted odds ratio
AUC: Area under the curve
BMI: Body mass index
COR: Crude odds ratio
ELISA: Enzyme linked immunosorbent assays
HADS-D: Hospital anxiety and depression scale-depression subscale
HbA1c: Hemoglobin A1c
HDL-cholesterol: High-density lipoprotein cholesterol
LAS: Left anterior descending artery stenosis
LDL-cholesterol: Low-density lipoprotein cholesterol
MMP: Matrix metalloproteinase
ROC: Receiver operating characteristics curve
TIMP: Tissue inhibitors of metalloproteinases
T1D: Type 1 diabetes
TIA: Transient ischemic attack
WC: Waist circumference
